# Optimal Conservative Management Resolves Refractory Hypoxemia in Patient with Right Myocardial Infarction Complicated by PFO-Induced Shunting

**DOI:** 10.14797/mdcvj.1191

**Published:** 2023-03-27

**Authors:** Suryono, Naesilla, Pipiet Wulandari, Dwi Ariyanti, Aditha Satria Maulana, Narendra Wahyu Junior, Hazbina Fauqi Ramadhan

**Affiliations:** 1 Dr. Soebandi General Hospital; 2 University of Jember, Jember, Indonesia; 3 Airlangga University, Surabaya, Indonesia

**Keywords:** right ventricular infarction, right-to-left intracardiac shunt, patent foramen ovale, hypoxemia

## Abstract

Inferior myocardial infarction is often accompanied by infarction of the right ventricle (RV). Uncommon RV infarction cases with patent foramen ovale (PFO) shunt, leading to severe persistent hypoxemia even without any pulmonary embolism involvement and often requiring invasive intervention, have been documented previously. We report a patient with RV infarction and right-to-left shunt via PFO who improved with only early revascularization and optimal standard treatment. This condition may not necessitate any invasive intervention if it is treated and monitored per standard procedures. Clinicians should consider the possibility of a right-to-left shunt in patients with RV infarction and persistent hypoxemia to implement appropriate therapeutic interventions.

## Introduction

Right ventricular (RV) ischemia accounts for complications in inferior myocardial infarctions (MIs) in up to 50% of cases.^1^,^2^ A higher risk of hemodynamic, electrophysiologic, and mechanical problems and an increased risk of in-hospital mortality have been linked to RV involvement. One rare mechanical complication of RV ischemia involves patent foramen ovale (PFO) and the associated right-to-left shunt leading to refractory hypoxia.^1^,^3^ We report a case of PFO disclosure following an inferior-RV infarction and highlight the importance of early and optimal pharmacological management.

## Case

**Figure 1:**
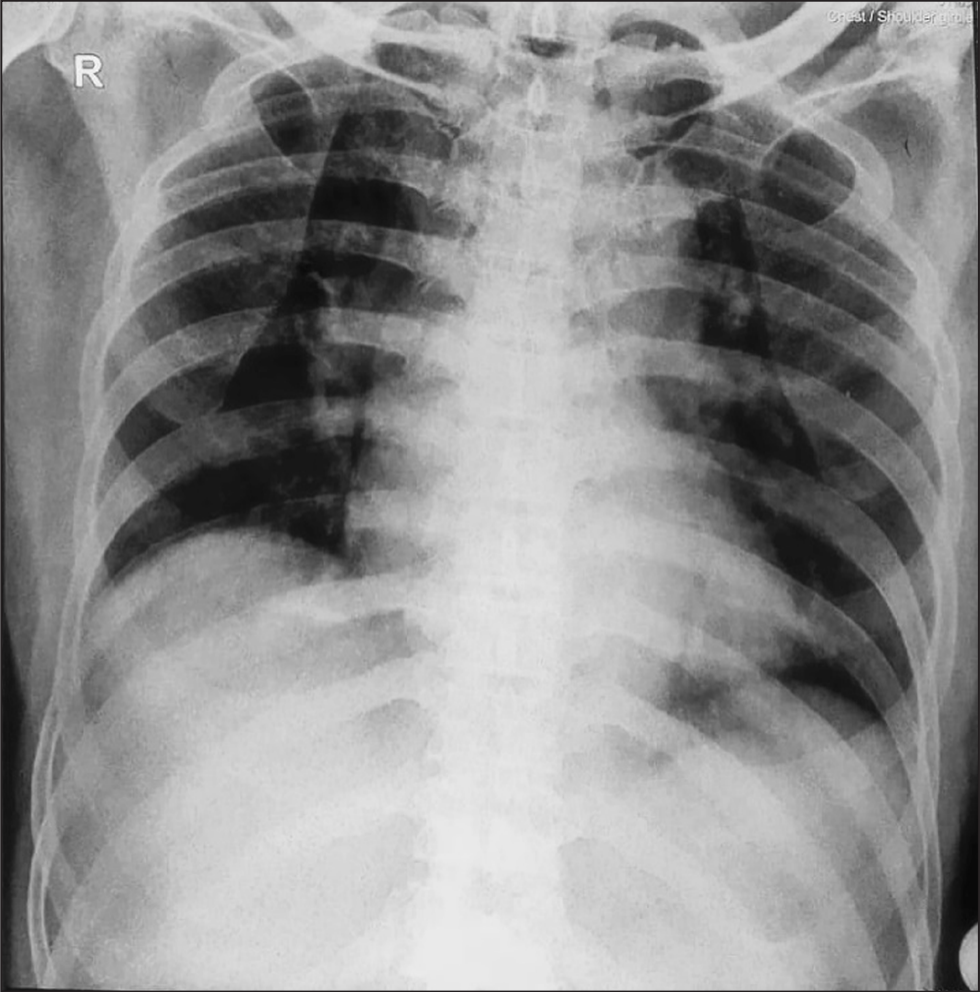
Chest radiograph at emergency department upon patient arrival. The radiograph showed clear lung fields.

A 67-year-old Indonesian man arrived in the emergency room 6 hours after the onset of chest pain. On arrival, he was weak but conscious, with severe hypotension (blood pressure was 70/50 mm Hg). He was bradycardic with a heart rate of 30 beats per minute and peripheral oxygen saturation (SaO_2_) of 94%. His extremities were cold and clammy. He was actively smoking without other reported cardiac risk factors (diabetes mellitus, hypertension, or familial history). He also denied any history of significant symptoms of deep vein thrombosis, such as leg pain. Physical examination revealed clear lungs with no murmurs or other sounds. A 12-lead electrocardiogram (ECG) discovered ST elevations in inferior leads (II, III, and aVF) supporting acute inferior MI diagnosis. Right-sided ECG confirmed the RV infarction (ST-elevations in leads V4R-V6R). Chest radiography indicated no evidence of congestion (Figure 1). Laboratory results were unremarkable.

**Figure 2:**
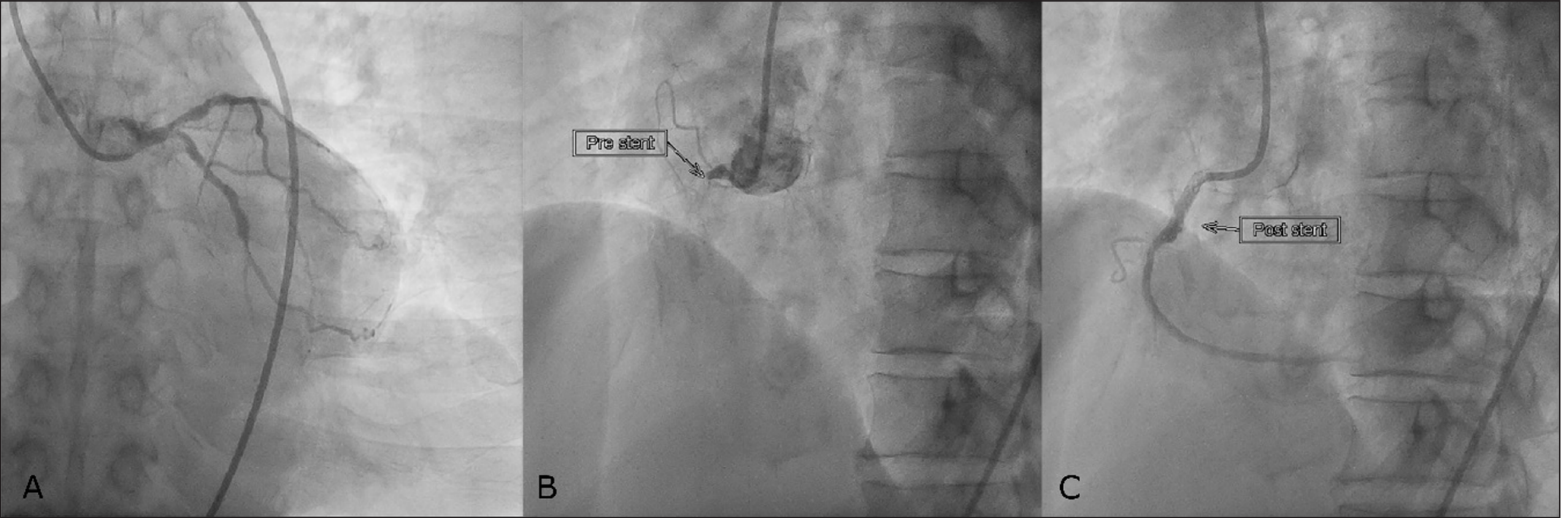
Coronary angiography. The angiogram showed multiple stenoses in (A) left coronary system and (B) proximal total occlusion of the right coronary artery, which (C) successfully restored post stenting.

His blood pressure improved to 100/70 mm Hg following dobutamine administration. The patient immediately received aspirin and clopidogrel per standard guidelines. Once stabilized, he was transferred to the catheterization lab for coronary angiogram and primary percutaneous cardiac intervention (PCI), which took place 7 hours after onset of chest pain. Total proximal occlusion of the right coronary artery was confirmed, whereas the left coronary arterial system presented with multiple stenoses in the left circumflex artery and left anterior descending artery. The flow was restored following balloon angioplasty and the placement of a 3.0-mm × 1.5-mm sirolimus-eluting coronary stent (TIMI grade III; Figure 2).

**Figure 3:**
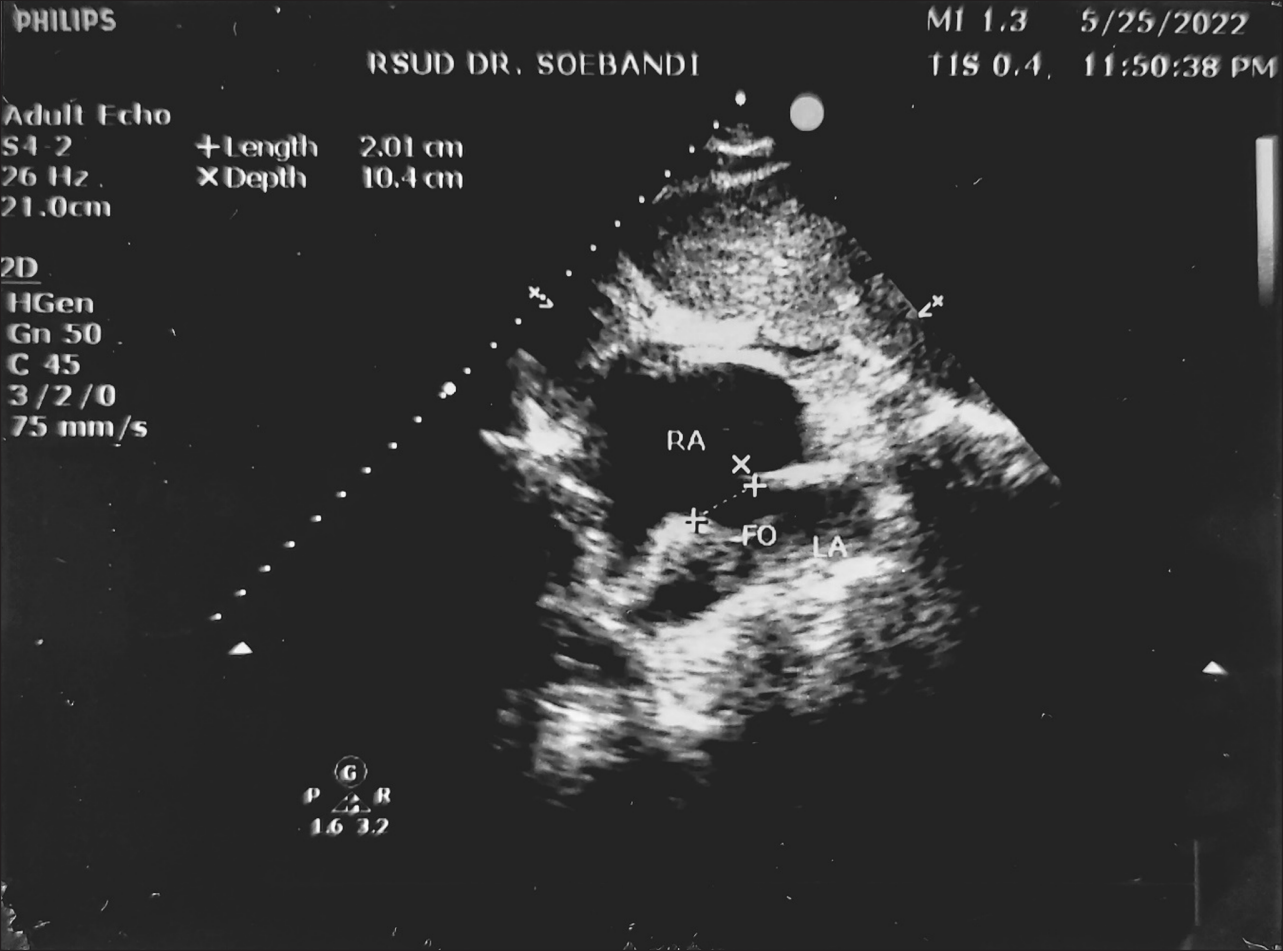
Emergency transthoracic echocardiogram. There was a 2-cm flap-like opening indicating patent foramen ovale.

The patient’s hemodynamics were monitored and stable in the cardiac intensive care unit when he suddenly had a generalized seizure episode that lasted approximately 5 seconds. Post-seizure heart monitoring still showed bradycardia. His oxygen saturation dropped to 81% and his blood pressure continued to fall, reaching a low of 85/60 mm Hg. There was no evidence of neurological symptoms. Physical examination proved clear lung auscultation with elevated jugular venous distention. Along with hypotension, these signs led the physician to perform emergency transthoracic echocardiography to confirm RV dysfunction and exclude differential diagnoses, such as mechanical complications. The initial ECG showed normal left ventricular (LV) function with diminished RV function (indicated by tricuspid annular plane systolic excursion = 0.765 cm) and a flap-like PFO in the atrial septum with a 2-cm opening. A right-to-left shunt was observed and positively confirmed with a bubble test (Figure 3). Arterial blood gas assessment revealed a partial oxygen pressure of 40 mm Hg in room air, confirming the presence of type I respiratory failure. He underwent a chest computed tomography, which showed an abnormal interatrial contrast flow confirming a PFO and aortic sclerosis, with no evidence of thrombus or emboli in either the right atrium, RV, or pulmonary artery.

The patient was closely monitored and responded to the current regimen (blood pressure improved to 95/70 mm Hg with dobutamine and fluid therapy). Nevertheless, hypoxemia persisted for 3 days despite high-flow oxygen supplementation (oxygen saturation fluctuated between 81% and 89%) before gradually increasing on day 4 and reaching a normal value of 98% on day 6.

Predischarge transesophageal echocardiogram on day 7 showed that the PFO flap was closed, with no sign of a right-to-left shunt, confirmed by negative results on the cough maneuver and bubble test (Video 1). He stabilized, reported no symptoms (oxygen saturation of 98% and blood pressure of 125/80 mm Hg), and was discharged the next day. He reported no complaints during the follow-up visits.

**Video 1:** Pre-discharge imaging. Transesophageal echocardiogram demonstrates the closed position of patent foramen ovale flap with negative bubble test; see also at https://youtu.be/Q9_kkSG98fw.

## Discussion

The foramen ovale is an important part of the fetal circulatory system that closes permanently in 70% of individuals following birth. However, in 30% of cases, it remains a silent PFO and transforms into a flap-like valve. This flap prevents left-to-right shunt; once the right atrial pressure surpasses the left, it creates a gap, causing right-to-left shunting.^4^,^5^

Similar to previous reports, the patient developed characteristic persistent hypoxemia following MI and PCI procedures.^2^,^6^ After successful PCI, the patient achieved transient hemodynamic stabilization before having a seizure followed by severe hypoxemia and blood pressure drop. We believe the seizure was associated with the abrupt drop in oxygen supply without leaving any neurological sequelae. The management, including high-flow oxygen with a non-rebreather mask, increased dose of the inotropic support, and optimal fluid management initially only corrected his blood pressure and hemodynamics while he still presented refractory hypoxemia. Suspicion of cardiac failure and PFO shunt led to emergency echocardiography that revealed significant RV dysfunction and the right-to-left shunt from a flap-like PFO.

Hypoxemia due to PFO is a rare complication of RV infarction.^7^ Other than RV infarction, persistent right-to-left shunt via PFO has been reported in association with RV dysfunction or elevated pulmonary pressure that led to RV volume overload, including chronic obstructive pulmonary disease, tricuspid valve or pulmonary valve regurgitation, RV infarction, or recurrent pulmonary embolism.^8^,^9^ In this patient, a PFO was identified for the first time in his life following inferior and right MI without any signs or symptoms of other causes. Few case reports have documented PFO in RV dysfunction cases specifically induced by MI; however, the number of reported cases is significantly lower compared to PFO with pulmonary embolism-related MI.

This right-to-left shunt may cause oxygen-resistant systemic hypoxemia. The right ventricle is generally infarct-resistant because it consumes a lower oxygen supply and has a higher oxygen reserve with a direct perfusion supply. The RV is expected to have a high degree of recovery after extended occlusion. However, it has slow functional recovery that may increase in-hospital mortality. Of the patients who present with RV branch occlusion, 40% to 50% undergo RV dysfunction. As the injury reduces cardiac compliance, the right end-diastolic and atrial pressures increase, exceeding the left atrial pressure. The right atrium dilates with increased right-to-left gradient and eventually opens the formerly silent PFO, whereby the patient develops a right-to-left shunt. Because this complication is uncommon, the physician must consider a differential diagnosis of right-to-left shunt via PFO in prolonged refractory hypoxemia cases following RV infarction, particularly if pulmonary embolism has been excluded.^2^,^7^

Treatment focusing on optimal volume status, inotropic support, physiologic rhythm restoration, and coronary reperfusion is still the mainstay.^6^ Inhaled nitric oxide was shown in two previous studies to increase cardiac function and systemic oxygenation by reducing RV preload.^10^,^11^ However, other studies stated that the inhaled nitric oxide only presented temporary improvement and did not impose any meaningful improvement.^5^,^12^ Inhaled nitric oxide has the potential to lower systemic afterload, tending to decrease left atrial pressure and worsen the right-to-left shunt. Furthermore, nitrate tolerance may develop before RV function recovery is achieved and will require more definitive shunt closure.^13^ Therefore, early revascularization and watchful waiting with optimal medication is still the first-line treatment; both were received by the patient in this case. Reperfusion of the culprit lesion in acute myocardial ischemia should be engaged as soon as feasible to achieve the best outcome.

Regarding the closure of the PFO, several studies performed definitive closure using occluder devices due to worsening and refractory hypoxemia despite optimal conservative treatment. A previous study debated the benefit of occlusion. Two reported cases achieved hemodynamic and oxygenation normalization under inotropic support; these cases were deemed inappropriate for PFO occlusion intervention. In addition, the occlusion of a PFO may worsen LV filling and increase RV filling in cardiogenic shock due to RV failure setting. The closure of the PFO must be considered in persistent clinically significant shunts or cases without any recovery of RV function.^6^,^9^,^12^,^14^,^15^

## Conclusion

This case report demonstrates the importance of evaluating PFO-induced right-to-left shunt as a cause of refractory hypoxemia in patients with right MI. This diagnosis prompts the need for earlier revascularization and optimal conservative treatment to achieve the best outcome.
